# Post-natal Growth Retardation Associated With Impaired Gut Hormone Profiles, Immune and Antioxidant Function in Pigs

**DOI:** 10.3389/fendo.2019.00660

**Published:** 2019-09-26

**Authors:** Ming Qi, Bie Tan, Jing Wang, Simeng Liao, Jianjun Li, Yanhong Liu, Yulong Yin

**Affiliations:** ^1^Laboratory of Animal Nutritional Physiology and Metabolic Process, Key Laboratory of Agro-Ecological Processes in Subtropical Region, National Engineering Laboratory for Pollution Control and Waste Utilization in Livestock and Poultry Production, Institute of Subtropical Agriculture, Chinese Academy of Sciences, Changsha, China; ^2^University of Chinese Academy of Sciences, Beijing, China; ^3^College of Animal Science and Technology, Hunan Agricultural University, Changsha, China; ^4^Department of Animal Science, University of California, Davis, Davis, CA, United States

**Keywords:** post-natal growth retardation, hormone secretion, appetite, immune response, antioxidant capacity, blood parameters

## Abstract

The factors that cause post-natal growth retardation (PGR) in pigs are complicated; however, metabolic and immune system impairment seem to be involved. The purpose of this study was to investigate the changes of blood parameters, hormone profiles, antioxidant capacity, and immune responses in PGR pigs. Blood and small intestinal mucosa samples were collected from 42-days-old PGR and healthy pigs. The results showed that compared with the healthy group, the relative weight of spleen and kidney were greater, but the liver was lighter in PGR pigs (*P* < 0.05). The PGR pigs had increased serum alanine transaminase, urea nitrogen, blood ammonia, IgG, and complement 4, but decreased glucose and albumin (*P* < 0.05). The higher levels of serum leptin (LEP) and thyroxin (T4), and the lower levels of insulin-like growth factor-1 (IGF-1), 5-hydroxytryptamine (5-HT), somatostatin (SS), and agouti gene-related protein (AgRP) were observed in PGR pigs (*P* < 0.05). Consistent with the serum levels of hormones, the mRNA levels of gut hormones and their receptors were also altered in intestinal mucosa from PGR pigs (*P* < 0.05). The PGR pigs exhibited higher plasma concentrations of interleukin-1β (IL-1β), IL-6, IL-8, and transformed growth factor beta (TGFβ) (*P* < 0.05). However, the mRNA expressions of several cytokines were lower in the small intestinal mucosa of PGR pigs (*P* < 0.05). Abnormal antioxidant indexes in serum of PGR pigs were observed, which was in accordance with the reduced mRNA expression of several anti-oxidative genes in the small intestinal mucosa of PGR pigs (*P* < 0.05). These data demonstrate that an abnormal gut hormone system, immune dysfunction, and decreased antioxidant capacity may contribute to PGR in pigs. These changes could provide a valuable target in the regulation of post-natal growth retardation in animals and humans.

## Introduction

Post-natal growth retardation (PGR) piglet refers to a piglet that has failed to achieve its genetically determined growth potential (1). PGR is associated with lifelong consequences beyond reduced weight, including metabolic disturbance and impaired immune function (2). The intrauterine growth restriction (IUGR) pigs also exhibit lower diversity and different taxonomic abundances of gut microbiota (3). Although possible causes of PGR have been discussed, such as placental insufficiency (4), pathogen burden (5), gut mucosal barrier dysfunction (6), parental genetic disruption (7), and abnormal maternal lactocrine programming (8), the question remains largely unsolved as to the assessment of the common markers between PGR pigs and pigs with normal growth. It is important to seek the possible intervening targets to enhance growth in poorly performing piglets, ensuring a good growth rate post-weaning (9, 10).

Blood profiles, such as acute-phase proteins, have been used to assess the herd health, immune status, and growth potential in pigs (11, 12). Marked differences are observed in serum glucose concentration between growth-retarded children and healthy children, and some types of amino acids are significantly reduced in fetal growth restriction children (13). The levels of multiple hormones, such as growth hormone (GH), thyroxin (T4), and leptin (LEP) were also altered in IUGR children and piglets (14, 15). These anabolic hormones play important roles in many biological activities including cell survival, organ maturation, anti-inflammatory, and antioxidant (16, 17). Insulin sensitivity in IUGR pigs can be improved with dietary methionine restriction (18). Therefore, the disturbance of their secretion or function during post-natal development in PGR induces long-term changes in tissue growth and function, with persistent detrimental effects (15). IUGR pigs were also reported to have impaired antioxidant capacity and disorder of the immune system (19, 20). The increased serum levels of proinflammatory cytokines, such as interleukin-8 (IL-8) reflects the severe inflammatory response in pigs (21, 22).

Therefore, we hypothesized that changes of blood profiles, abnormal gut hormone secretion, immune dysfunction, and decreased antioxidant capacity may contribute to PGR in pigs. The present study was conducted to compare the differences in blood parameters, gut hormone profiles, antioxidant capacity, and immune responses between PGR pigs and healthy pigs, which could provide a valuable target in the regulation of PGR in animals and humans.

## Materials and Methods

### Animals and Experimental Design

This study was conducted in accordance with the guidelines of the Institute of Subtropical Agriculture, Chinese Academy of Sciences. All experimental protocols were approved by the animal ethical committee of the Institute of Subtropical Agriculture, Chinese Academy of Sciences (2013020).

Duroc × Landrace × Large Yorkshire crossbred pigs with the same paternal origin weaned at 21 days of age were used in the present study. All pigs were housed in pens with hard plastic slatted flooring and had 24 h *ad libitum* access to feed and water. The room temperature was kept at 26 ± 1 °C, and the humidity was controlled between 50 and 60%. Pigs were fed the same commercial feeds which were formulated according to the recommended nutrient requirements of National Research Council (2012) (23). At 42 d of age, six PGR pigs (BW 5.40 ± 0.38 kg) and six healthy pigs (Control) (BW 11.01 ± 0.40 kg) pair-matched by litter were selected for sampling. Pigs with a BW of <70% of average BW were regarded as PGR, and there were no obvious characteristics of the disease or injury. After overnight fasting, blood samples were obtained from the jugular vein in the morning (18). Approximately 10 mL of blood from the jugular vein was collected in aseptic capped tubes containing 150 U of sodium heparin, and another 10 mL of blood were routinely collected. Serum and plasma samples were obtained by centrifugation at 2,000 × g for 10 min at 4°C. These samples were immediately stored at −80°C for analyses of biochemical profile, antioxidant capacity, hormone profiles, and cytokine production. All pigs were anesthetized with sodium pentobarbital (20 mg/kg BW) and killed by jugular puncture. The liver, kidney, spleen, heart, and lung were obtained and weighed. The relative weight of each organ was calculated as the organ weight divided by the BW (g/kg). Samples of the jejunal and ileal mucosa were scraped and immediately snap-frozen in liquid nitrogen and stored at −80°C for RNA extraction.

### Serum Biochemical Indexes Assays

Immunoglobulins (IgG and IgM), as well as biochemical indicators (total protein, albumin, etc.) were measured using an instrument (Biochemical Analytical Instrument, Beckman CX4, Beckman Coulter Inc., Brea, CA) and commercial kits (Sino-German Beijing Leadman Biotech Ltd., Beijing, China).

### Determination of Serum Hormone

Serum concentrations of GH, T4, LEP, 5-hydroxytryptamine (5-HT), somatostatin (SS), insulin (INS), insulin like growth factor-1 (IGF-1), glucagon-like peptide 1 (GLP-1), agouti gene-related protein (AgRP), and proopiomelanocortin (POMC) were determined using ELISA kits in accordance with the manufacturer's instructions (Meimian industrial Co., Ltd., Jiangsu, China).

### Determination of Plasma Cytokines

Plasma concentrations of interleukin-1β (IL-1β), IL-6, IL-8, IL-10, IL-12, tumor necrosis factor alpha (TNFα), transformed growth factor beta (TGFβ), and interferon gamma (IFNγ) were measured using commercially available swine enzyme-linked immunosorbent assay (ELISA) kits according to the manufacturer's instructions (Meimian industrial Co., Ltd., Jiangsu, China).

### Serum Antioxidant Capacity

The serum antioxidant indices, including glutathione peroxide (GSH-PX), glutathione S-Transferases (GST), total antioxidant capacity (T-AOC), superoxide dismutase (SOD) activities, malondialdehyde (MDA), nitric oxide (NO) content, and total nitric oxide synthase (TNOS) were measured using commercial kits (Jiancheng Bioengineering Institute, Nanjing, China) according to the manufacturer's instructions.

### Real-Time Quantitative RT-PCR

Total RNA was isolated from the liquid nitrogen-pulverized intestinal mucosa samples with the TRIZOL reagent (Invitrogen, Carlsbad, CA, USA) according to the manufacturer's instructions and quantified by electrophoresis on 1% agarose gel with the measurement of optical density at 260 and 280 nm. Synthesis of the first strand (cDNA) was performed with 5 × PrimeScript Buffer2 and PrimeScript reverse transcriptase Enzyme Mix 1 (Takara Biotechnology (Dalian) Co., Ltd, Dalian, China). Primers were designed with Primer 5.0 (PREMIER Biosoft International, Palo Alto, CA, USA) according to the gene sequence of the pig to produce an amplification product, as described previously (24). The primers used to amplify genes are shown in Table 1. β*-actin* was used as a housekeeping gene to normalize target gene transcript levels (21). The resulting cDNA was diluted and used as a PCR template to evaluate gene expression. The reaction was performed in a volume of 10 μL (LightCycler^®^ 480 Real-Time PCR System, Roche, Switzerland). 1 μL cDNA template was added to a total volume of 10 μL containing 5 μL SYBR Premix Ex Taq II (Takara, Dalian, China), 3.2 μl distilled H2O, and 0.4 μmol/L each of forward and reverse primer. Reactions were incubated in a 384-well optical plate (Roche, Switzerland). We used the following protocol: (i) predenaturation program (30 s at 95°C), (ii) amplification and quantification program repeated 40 cycles (5 s at 95°C and 20 s at 60°C for annealing), and (iii) melting curve program (60–95°C with a heating rate of 0.1°C per second and fluorescence measurement). All samples were tested in triplicate. The relative expression levels of the selected genes normalized against the reference gene (β*-actin*) were calculated by using the 2^−ΔΔCt^ method (25). No expression changes of β*-actin* were observed in the intestinal mucosa among the two groups (data not shown). Data are expressed as the relative values to those for healthy pigs.

**Table 1:** Primers used for quantitative reverse transcription-PCR.

**Gene**	**Gene Bank No**.	**Sequence (5′-3′)**	**Product length, bp**
*β-actin*	XM_0031242803	F:CTGCGGCATCCACGAAACT	147
		R:AGGGCCGTGATCTCCTTCTG	
*IL-1β*	NM_214055.1	F:CAGCCATGGCCATAGTACCT	216
		R:CCACGATGACAGACACCATC	
*IL-4*	NM_214340.1	F:CCCGAGTGTCAAGTGGCTTA	122
		R:TGATGATGCCGAAATAGCAG	
*IL-6*	NM_001252429.1	F:GGCAAAAGGGAAAGAATCCAG	87
		R:CGTTCTGTGACTGCAGCTTATCC	
*IL-8*	NM_213867.1	F:GCTCTCTGTGAGGCTGCAGTTC	79
		R:AAGGTGTGGAATGCGTATTTATGC	
*IL-10*	NM_214041.1	F:GGGCTATTTGTCCTGACTGC	105
		R:GGGCTCCCTAGTTTCTCTTCC	
*IFNγ*	NM_213948.1	F:TTCAGCTTTGCGTGACTTTG	121
		R:GGTCCACCATTAGGTACATCTG	
*TLR4*	NM_001113039.1	F:CAGATAAGCGAGGCCGTCATT	113
		R:TTGCAGCCCACAAAAAGCA	
*MyD88*	NM_001099923	F:GTGCCGTCGGATGGTAGTG	65
		R:TCTGGAAGTCACATTCCTTGCTT	
*Nrf2*	XM_005671981.3	F:CACCACCTCAGGGTAATA	125
		R:GCGGCTTGAATGTTTGTC	
*HO-1*	NM_001004027.1	F:AGCTGTTTCTGAGCCTCCAA	130
		R:CAAGACGGAAACACGAGACA	
*NQO1*	NM_001159613.1	F:CCAGCAGCCCGGCCAATCTG	160
		R:AGGTCCGACACGGCGACCTC	
*Akt*	NM_001159776.1	F:TGTGGCAGGATGTGTATGAGA	188
		R:GTAGGAGAACTGGGGGAAGTG	
*mTOR*	XM_003127584.6	F:TTGTTGCCCCCTATTGTGAAG	61
		R:CCTTTCGAGATGGCAATGGA	
*4EBP1*	NM_001244225.1	F:CCGGAAGTTCCTAATGGAGTGT	125
		R:GGTTCTGGCTGGCATCTGT	
*P70S6K*	XM_003131671.5	F:GGAAACAAGTGGAATAGAGCAGATG	65
		R:TTGGAAGTGGTGCAGAAGCTT	
*GHRL*	NM_213807.2	F: CAAGAAGCCAGCAGCCAAAC	290
		R: GAAGCCAGGTGAGCCCTTAG	
*AgRP*	NM_001011693.1	F: GCAGGCCGAGGCCAA	57
		R: CGTGCCTTGCGTCCTTC	
*SS*	NM_001009583.1	F: CTCTCCATCGTCCTGGCTCT	159
		R: GTTCTCTGTCTGGTTGGGTTCAG	
*IGF-1*	NM_214256.1	F: GACGCTCTTCAGTTCGTGTG	141
		R: CTCCAGCCTCCTCAGATCAC	
*IGF-1R*	NM_214172.1	F: CAGTCCTAGCACCTCCAAGC	134
		R: GTCTTCGGCCACCATACAGT	
*GHR*	NM_214254.2	F: TTCACCAAGTGCCGTTCA	154
		R: AATCAGGGCACTCTTTC	
*INSR*	XM_021083940.1	F: GGCATGGTGTACGAGGGAAA	124
		R: AGGCCTCGTTGAGAAACTCG	
*SERT*	XM_021067520.1	F: GGCTTGGAGGGGGTGATCA	60
		R: GCGCTTGGACCAGAAGT	
*5-HTR4*	NM_001001267.1	F: ACAGGAACAAGATGACCCCT	277
		R: AGGAGGAACGGGATGTAGAA	
*CCK*	NM_214237.2	F: GGCCAGATACATCCAGCAG	122
		R: AATCCATCCAGCCCATGTAG	
*GLP-1R*	NM_001256594.1	F: CACAGGCTTGTTCTGCAACC	414
		R: AAGACGGACAGTGCTCGAAG	

### Statistical Analyses

All statistical analyses were performed by the independent sample *t*-test using SPSS software 20.0 (SPSS Inc., Chicago, IL). *P*-values < 0.05 were taken to indicate statistical significance.

## Results

### BW and Relative Weight of Visceral Organs

As shown in Table 2, the average BW of PGR was significantly lighter than that of age-matched healthy pigs (*P* < 0.01). Absolute weight of all organ samples was significantly lower for PGR pigs compared to healthy pigs (*P* < 0.01). Compared with healthy pigs, the relative weights of the spleen and kidney were increased but the relative weight of the liver was decreased in PGR pigs (*P* < 0.05).

**Table 2:** The body weight and viscera weights of healthy (Control) and post-natal growth retardation (PGR) pigs (*n* = 6)^a^.

**Item**	**Control**	**PGR**	**SEM**	***P*-value**
BW, kg	11.01	5.40	0.57	<0.01
**Heart**				
Weight, g	54.62	27.26	3.40	<0.01
Relative weight, g/kg	4.95	5.24	0.23	0.257
**Liver**				
Weight, g	328.61	122.16	15.12	<0.01
Relative weight, g/kg	29.92	23.71	1.72	<0.01
**Spleen**				
Weight, g	22.67	14.00	2.48	<0.01
Relative weight, g/kg	2.05	2.60	0.19	0.015
**Lung**				
Weight, g	160.54	90.09	3.78	<0.01
Relative weight, g/kg	14.69	17.18	1.53	0.136
**Kidney**				
Weight, g	66.58	35.96	4.18	<0.01
Relative weight, g/kg	5.78	6.92	0.39	0.016

a *BW, body weight. The relative weights of viscera were calculated as the organ weight divided by BW (g/kg)*.

### Serum Biochemical Indexes

The serum concentrations of IgG and complement 4 (C4) were higher in PGR pigs as compared to healthy pigs (*P* < 0.05). An increase in serum concentrations of alanine transaminase, blood urea nitrogen, and blood ammonia but a decrease in albumin, alkaline phosphatase, and glucose were observed in PGR pigs (*P* < 0.05). There were no differences in total protein, aspartate aminotransferase, lactic dehydrogenase, and lactate between the two groups (*P* > 0.05) (Table 3).

**Table 3:** Serum biochemical indexes in healthy (Control) and post-natal growth retardation (PGR) pigs (*n* = 6).

**Item**	**Control**	**PGR**	**SEM**	***P*-value**
IgG, g/L	1.14	1.83	0.22	0.011
IgM, g/L	0.54	0.57	0.09	0.787
Complement 3, mg/L	20.00	20.00	5.00	0.515
Complement 4, mg/L	20.00	30.00	3.00	0.011
Total protein, g/L	49.70	48.98	1.40	0.617
Albumin, g/L	27.50	21.94	1.33	0.002
Alkaline phosphatase, U/L	357.80	107.20	26.83	<0.01
Alanine transaminase, U/L	36.27	58.06	4.21	<0.01
Aspartate aminotransferase, U/L	52.60	80.00	13.36	0.067
Lactic dehydrogenase, U/L	803.00	859.50	38.20	0.170
Glucose, mmol/L	6.18	4.50	0.49	0.006
Blood urea nitrogen, mmol/L	2.02	2.32	0.10	0.014
Blood ammonia, μmol/L	414.98	547.13	38.51	0.006
Lactate, mmol/L	9.81	7.76	0.95	0.055

### Hormone Concentration in Serum

As shown in Table 4, the serum concentrations of IGF-1, 5-HT, and AgRP in PGR pigs were significantly lower than those of healthy pigs (*P* < 0.05). However, PGR significantly increased the levels of LEP, SS, and T4 in serum as compared to healthy pigs (*P* < 0.05). No significant difference was observed in serum concentrations of GH, INS, GLP-1, and POMC between the two groups (*P* > 0.05).

**Table 4:** Serum hormone concentrations in healthy (Control) and post-natal growth retardation (PGR) pigs (*n* = 6).

**Items**	**Control**	**PGR**	**SEM**	***P*-value**
GH, ng/mL	18.43	21.53	2.06	0.163
IGF-1, ng/mL	91.14	81.77	2.87	<0.01
5-HT, pg/mL	1,630.72	1,312.29	121.49	0.026
LEP, ng/mL	12.31	14.64	1.00	0.042
INS, mIU/L	27.99	30.65	2.68	0.344
T4, pmol/L	22.52	29.12	2.43	0.022
SS, pg/mL	55.32	73.32	6.40	0.018
GLP-1, pmol/L	12.23	13.12	1.23	0.489
AgRP, pg/mL	2,803.26	2,159.39	232.78	0.020
POMC, ng/mL	11.82	12.66	1.54	0.596

*GH, growth hormone; IGF-1, insulin like growth factor-1; 5-HT, 5-hydroxytryptamine; LEP, leptin; INS, insulin; T4, thyroxin; SS, somatostatin; GLP-1, glucagon-like peptide 1; AgRP, agouti gene-related protein; POMC, proopiomelanocortin*.

### Plasma Cytokines

**Figure 1:**
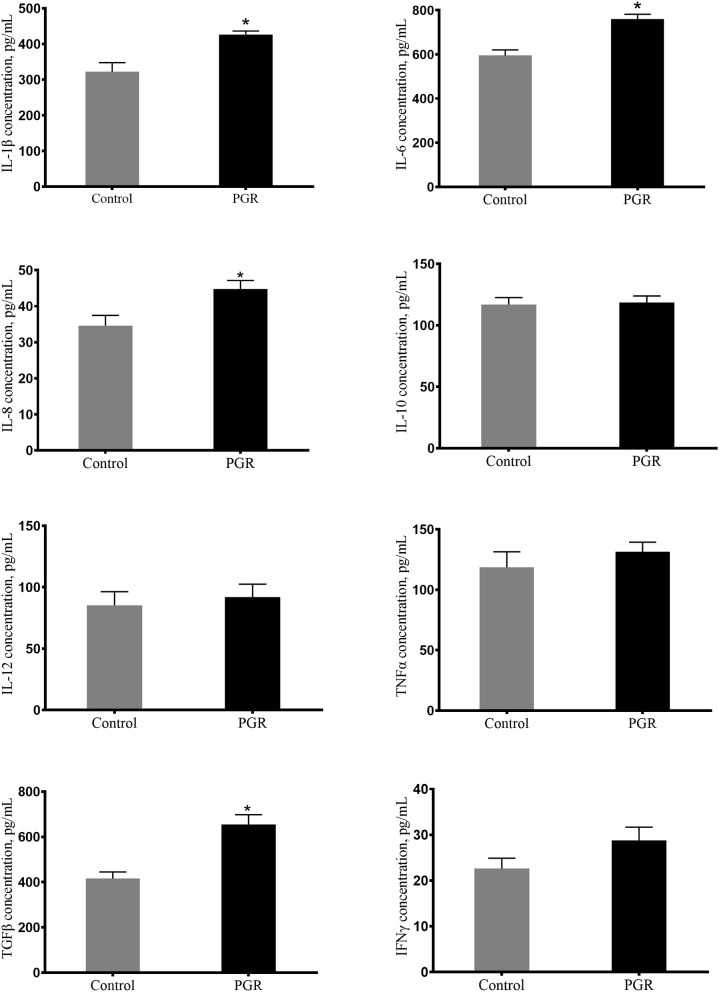
Concentrations of plasma cytokines in healthy pigs (Control) and post-natal growth retardation (PGR) pigs. Data are expressed as means ± SEM. *n* = 6. *P < 0.05 vs. healthy pigs.

Plasma concentrations of IL-6, IL-1β, IL-8, and TGFβ were significantly higher (*P* < 0.05) in PGR pigs in comparison to healthy pigs. And plasma concentrations of IL-10, IL-12, TNFα, and IFNγ were not affected in PGR (*P* > 0.05) (Figure 1).

### Serum Anti-oxidation Index

**Figure 2:**
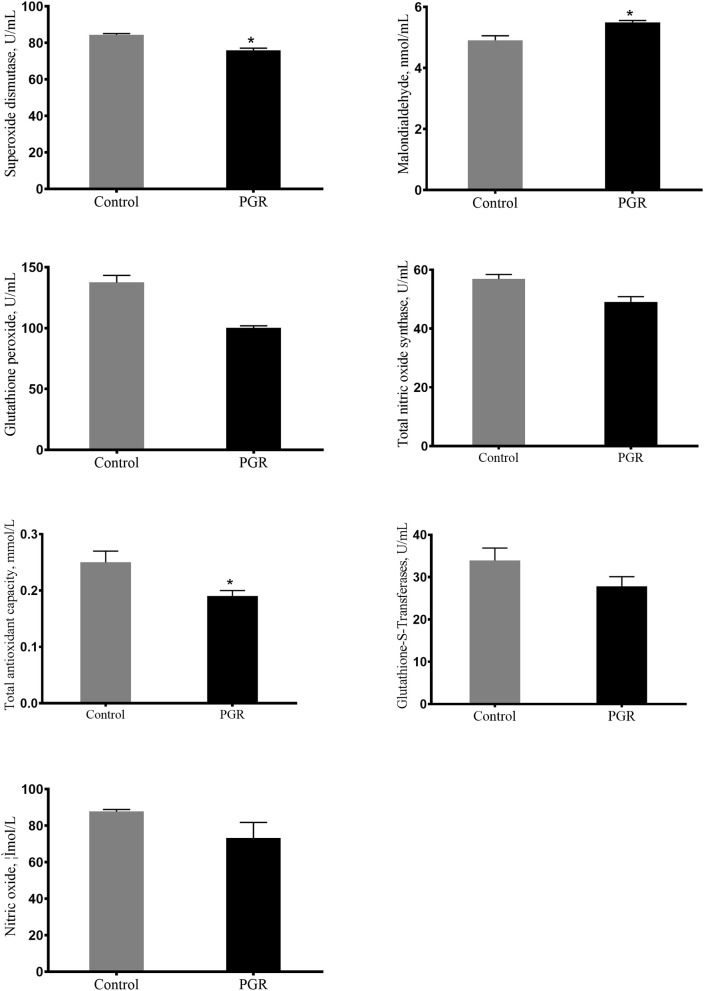
Serum antioxidant capacity of healthy pigs (Control) and post-natal growth retardation (PGR) pigs. Data are expressed as means ± SEM. *n* = 6. *P < 0.05 vs. healthy pigs.

The serum concentrations of T-AOC, SOD, MDA, GSH-PX, GST, NO, and TNOS are presented in Figure 2. Compared to healthy pigs, the serum concentrations of T-AOC, SOD, GSH-PX, and TNOS were decreased, but the concentration of MDA was significantly increased in PGR pigs (*P* < 0.05). However, there were no differences in the serum concentrations of GST and NO between the two groups (*P* > 0.05).

### Relative mRNA Expression of Intestinal Hormone-, Immune- and Antioxidant-Related Genes

**Figure 3:**
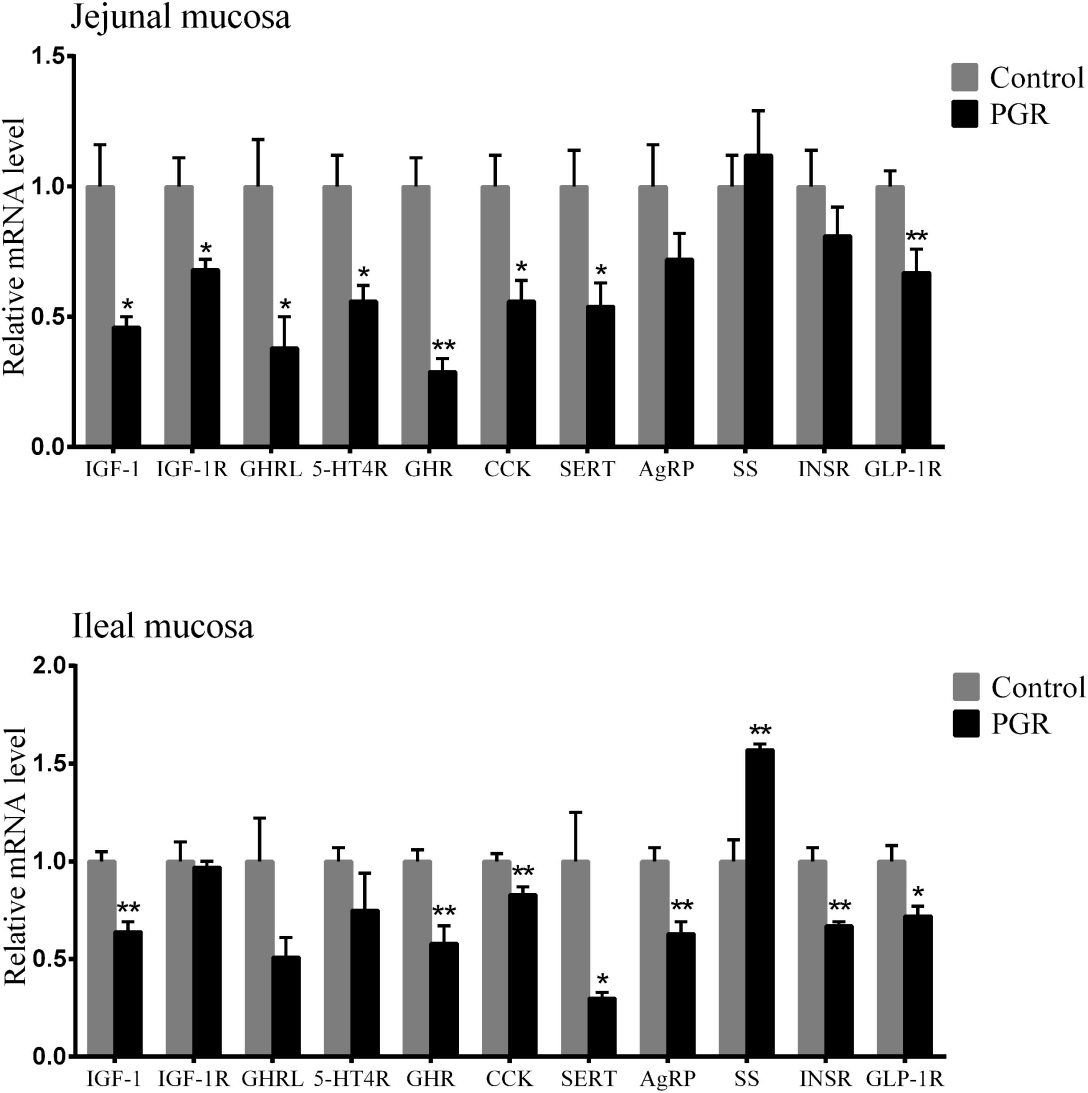
Relative mRNA abundances of hormone-related genes in the jejunal and ileal mucosa of healthy pigs (Control) and post-natal growth retardation (PGR) pigs. Data are expressed as means ± SEM, *n* = 6. *P < 0.05 vs. healthy pigs; **P < 0.01 vs. healthy pigs.

The relative mRNA expression of hormone-related genes is shown in Figure 3; PGR induced decreased mRNA levels of *IGF-1*, growth hormone receptor (*GHR*), cholecystokinin (*CCK*), serotonin transporter (*SERT*), and glucagon-like peptide 1 receptor (*GLP-1R*) in jejunal and ileal mucosa as compared to healthy pigs (*P* < 0.05). Compared to healthy pigs, the relative mRNA levels of insulin like growth factor-1 receptor (*IGF-1R*), ghrelin (*GHRL*), and 5-hydroxytryptamine receptor 4 (*5-HTR4*) were significantly lower in jejunal mucosa than in PGR pigs (*P* < 0.05), while *AgRP* and insulin receptor (*INSR*) were downregulated in ileal mucosa from PGR pigs (*P* < 0.01). However, PGR significantly increased *SS* levels in ileal mucosa as compared to healthy pigs (*P* < 0.01).

**Figure 4:**
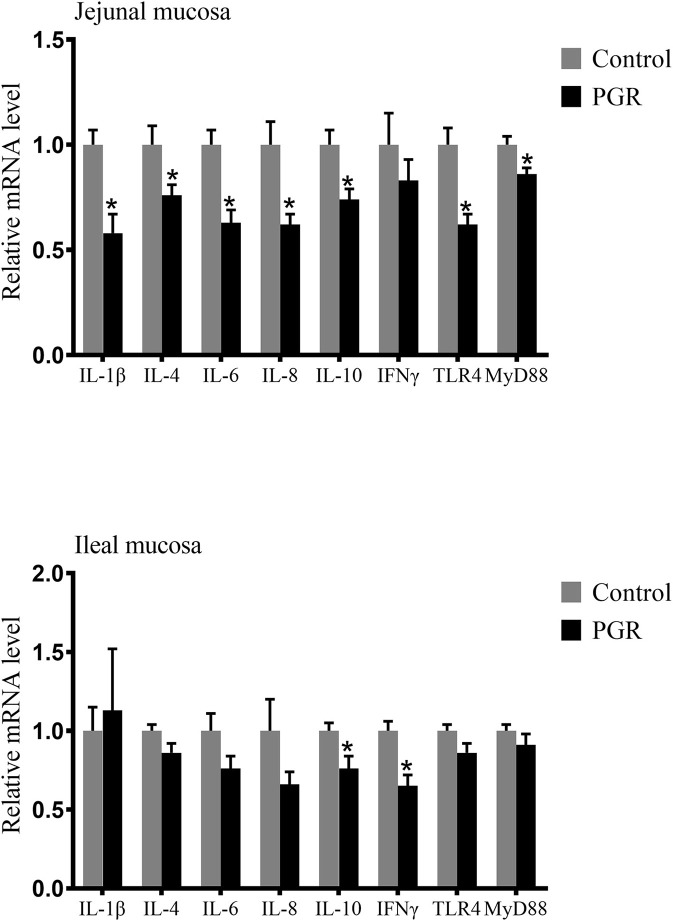
Relative mRNA abundances of cytokines in the jejunal and ileal mucosa of healthy pigs (Control) and post-natal growth retardation (PGR) pigs. Data are expressed as means ± SEM, *n* = 6. *P < 0.05 vs. healthy pigs.

As shown in Figure 4, the relative mRNA expressions of *IL-1*β, *IL-4, IL-6, IL-8, IL-10, IFN*γ, toll-like receptor 4 (*TLR4*), and myeloid differentiation primary response 88 (*MyD88*) in the jejunal mucosa and *IL-10* and *IFN*γ in the ileal mucosa were downregulated in PGR compared to those of healthy pigs (*P* < 0.05).

**Figure 5:**
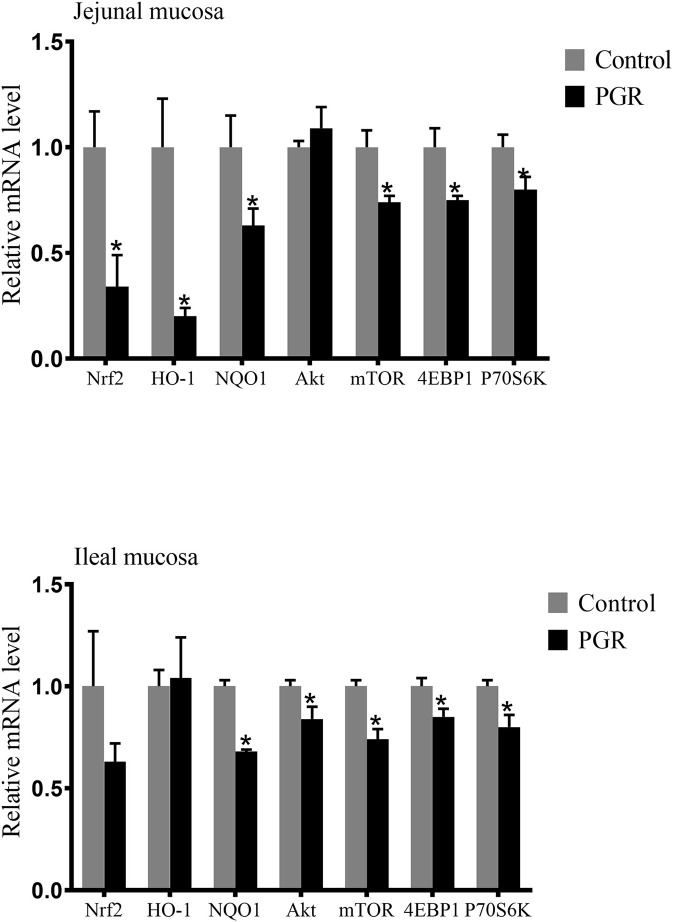
Relative mRNA abundances of antioxidant related genes and mTOR signaling pathway in the jejunal and ileal mucosa of healthy pigs (Control) and post-natal growth retardation (PGR) pigs. Data are expressed as means ± SEM, *n* = 6. *P < 0.05 vs. healthy pigs.

In the jejunal mucosa, the relative mRNA expressions of nuclear factor like-2 (*Nrf2*), heme oxygenase 1 (*HO-1*), NAD (P) H dehydrogenase 1 (*NQO1*), mechanistic target of rapamycin kinase (*mTOR*), eukaryotic translation initiation factor 4E binding protein 1 (*4EBP1*) and ribosomal protein S6 kinase beta-1 (*P70S6K*) were significantly decreased (*P* < 0.05) in PGR pigs compared to healthy pigs. In the ileal mucosa, PGR pigs showed lower expressions of serine-threonine protein kinase (*Akt*), *mTOR, 4EBP1, P70S6K*, and *NQO1* mRNA than in healthy pigs (*P* < 0.05) (Figure 5).

## Discussion

PGR, which causes reducing efficiency of feed utilization and increasing mortality (26), is common in the pig industry. Several studies have demonstrated that PGR exhibited poor growth performance compared with their heavier counterparts (2, 27). And it has been shown that PGR could lead to allometric growth of individual organs (28). The changes of relative weights of the liver, spleen, and kidney in PGR pigs were consistent with reports that the relative weight of the liver was decreased and kidney was increased in the IUGR lamb, and that of the spleen was increased in low birth weight piglets (2, 28). Many studies have shown that reduced relative weight of liver indicated a hepatic proinflammatory signal, and the increased relative weight of the spleen may be associated with high inflammation response, such as increased secretion of anti-inflammatory cytokines (29, 30). The injury of organs could be reflected by the changes of serum biochemical indexes, such as the blood urea nitrogen, ammonia, and C4 to some extent (31). Excessive inflammation activation may lead to liver impairment (32). The increased serum alanine transaminase in PGR pigs was proposed to reflect the decreased liver integrity, which agrees with the previous study that severe inflammation caused by lipopolysaccharide injection resulted in higher activity of serum alanine transaminase in mice (33). Additionally, glucose was significantly lower in serum from PGR pigs. Glucose is the primary source of energy for fetal growth, and energy restriction causes predictably growth reduction (28). As for the immunity, the PGR pigs exhibited higher plasma concentrations of IgG and some inflammatory cytokines. Increased serum IgG may indicate toxic or oxidative damage in organisms (34, 35).

The hormones play vital roles in regulation of growth and organ maturation. Shifts in hormone levels and their receptors' activity in PGR may induce persistent impacts on organ development and function (36). It has been reported that IUGR neonates showed the different hormone profile from non-IUGRs (36). In our study, the level of hormones and activity of their receptors were tested, and they—especially the gut hormones, such as 5-HT, LEP—were significantly changed between healthy and PGR pigs. This suggests that gut hormones may be one of the critical factors resulting in PGR. GH is a key factor that regulates growth and metabolism (37). The multifunctional actions of GH are determined by the abundance of GHR (37). No significant difference was observed in GH level between healthy and PGR pigs in our study, but decreased expression of *GHR* was observed in PGR pigs. This suggests that the abnormal expression of *GHR* but not the secretion of GH takes effects. In addition, many studies have demonstrated that IGF-1 played important roles during organ development in different animals. For example, the IGF-1 system is associated with neonatal growth in mice (38), liver development in rats (39), mammary maturation in swine (40), and bone remodeling in chickens (41). IGF-1 and GLP-1 also control the gastrointestinal tract development, inhibit the apoptosis of mammalian cells, and enhance anti-inflammatory and antioxidant capabilities (17, 36, 42). In our study, the serum concentration of IGF-1, and the mRNA expression of *IGF-1, IGF-1R*, and *GLP-1R* in intestinal mucosa, were decreased in PGR pigs as compared to healthy pigs. This agrees with the previous study that reduced plasma concentration of GLP-1 and the low circulating levels of IGF-1 were observed in IUGR pigs (17, 43). The deficiency in IGF-1 and GLP-1 leads to delayed maturation of intestinal mucosa, such as impaired intestinal barrier function and reduced activity of brush border enzymes, which may be one of the major causative factors in PGR (43, 44). Additionally, the elevated levels of T4 in serum from PGR pigs in our study may lead to increased oxidant production and mitochondrial oxidative damage (45). 5-HT is a vital regulatory factor in the gastrointestinal tract and other organ systems. The gut-derived 5-HT participates in various biological process, including intestinal motility and fluid secretion (46), immune responses (47), and bone development (48). The actions of 5-HT on enteric motor neurons are transduced by 5-HT receptors, such as 5-HT4R, which are majorly expressed in gastrointestinal tissues (49, 50). The SERT determines the final 5-HT availability, and its dysfunction is associated with various gut diseases (51). Our study showed that PGR reduced the serum level of 5-HT and mRNA expression of *5-HT4R* and *SERT* in intestinal mucosa. It is in accordance with the previous reports that altered 5-HT signaling resulted in both intestinal and extra-intestinal symptoms in gut disease, for example, the downregulated *SERT* and *5-HT4R* induced functional gut disorders, such as colitis (49, 52, 53). The intestinal hormones including LEP, INS, SS, AgRP, CCK, and GHRL, play important roles in regulating appetite via controlling digestion and satiety (54, 55). The appetite-stimulating effects of AgRP are inhibited by the secretion of LEP and activated by the GHRL (56). CCK mediates digestion by stimulating the activity of digestive enzymes to catalyze the nutrients, which is suppressed by SS secretion (57). In our study, we found elevated levels of LEP and SS but reduced levels of AgRP in serum, and mRNA levels of *CCK, INSR*, and *GHRL* in intestinal mucosa in PGR pigs as compared to healthy pigs. This agrees with a previous study that the level of *INSR* mRNAs tends to be lower in the intestinal mucosa of IUGR piglets compared to pigs with normal weight (58). Inhibited appetite and impaired digestive capability may contribute to PGR. The above findings may well explain the delayed maturation of intestinal mucosa in PGR pigs.

Pro-inflammatory cytokines (including IL-1β, IL-6, and IL-8) are necessary to initiate the inflammatory response during infection (59). In this study, the concentrations of IL-1β, IL-6, IL-8, and TGFβ in plasma were significantly higher in PGR pigs. It was coincident with the previous study that the activated immune response could lead to a high concentration of cytokines in plasma (21). However, the gene expression of several cytokines in the intestinal mucosa was lower in PGR pigs. This reflected the immaturity of the intestinal immune system in PGR pigs, which is consistent with a study that found IUGR piglets had decreased relative mRNA expression of cytokines in the small intestine (60).

Consistent with the impaired immune function of PGR pigs, PGR decreased antioxidant capacity. Oxidative stress may cause piglets' worsening health status and poor growth performance (61). At the cellular level, the exceeded reactive oxidative species (ROS) can lead to widespread damage to DNA, protein, and endogenous lipid (62). The increased concentration of MDA in serum was observed in PGRs, which are similar to the results of a previous study that the concentration of MDA was higher in the liver of IUGR piglets (19). SOD and GSH-PX are the main antioxidant enzymes that can be used to evaluate anti-oxidative capability in piglets, and they remove hydrogen peroxide via oxidizing reduced glutathione to oxidized glutathione (63). In this research, the activity of SOD and GSH-PX were significantly decreased in PGR pigs, which is in accordance with the previous results (2). TNOS and T-AOC reflect the levels of the non-enzymatic antioxidant defense system and antioxidant enzymes. PGR-induced oxidative injury was found to reduce the T-AOC and TNOS activity in serum, which is consistent with the findings of previous research that oxidative stress caused decreased activity of this enzyme (19, 64). In accordance with the lower antioxidant levels in serum from PGR pigs, lower expressions of anti-oxidation related genes including *Nrf2, NQO1*, and *HO-1* were also observed in PGR pigs. We further detected expressions of some critical genes involved in the Akt/mTOR pathway. The results showed that mRNA expressions of *Akt, mTOR, 4EBP1*, and *P70S6k* were down-regulated in intestinal mucosa from PGR pigs. This suggests that PGR may induce inhibition of intestinal epithelial cell proliferation and protein synthesis (65). The lower antioxidant capability may be explained by the abnormal mRNA expression of the Akt/mTOR pathway in PGR pigs (66).

In summary, PGR pigs had increased serum alanine transaminase, urea nitrogen, blood ammonia, IgG, and complement 4 but decreased glucose, and albumin. In addition, an impaired gut 5-HT pathway and abnormal secretion of hormones controlling digestion and gut maturation were observed in PGR pigs. Additionally, the PGR pigs exhibited higher plasma concentrations of IL-1β, IL-6, IL-8, and TGFβ, as well as lower serum T-AOC, SOD, GSH-PX, and TNOS. The mRNA expression of several anti-oxidative genes was also reduced in the small intestinal mucosa of PGR pigs. These results showed that PGR exhibited an abnormal hormone profile, immune dysfunction, and decreased antioxidant capacity, which could help to regulate post-natal growth retardation in animals and humans.

## Data Availability Statement

Data is available at NCBI under the accession number GSE136777.

## Ethics Statement

The animal study was reviewed and approved by the animal ethical committee of the Institute of Subtropical Agriculture, Chinese Academy of Sciences (2013020).

## Author Contributions

MQ, BT, and YY designed the experiments. MQ and JW conducted the experiments. SL and JL helped with animal experiments. MQ analyzed the data and wrote the original draft. YL and BT revised the manuscript. All authors read and approved the final manuscript.

### Conflict of Interest

The authors declare that the research was conducted in the absence of any commercial or financial relationships that could be construed as a potential conflict of interest.

## Abbreviations

PGR: post-natal growth retardation
BW: body weight
IL-1β: interleukin-1β
TGFβ: transformed growth factor beta
IgG: immunoglobulins G
TNFα: tumor necrosis factor alpha
IFNγ: interferon gamma
ELISA: enzyme-linked immunosorbent assay
GH: growth hormone
IGF-1: insulin like growth factor-1
5-HT: 5-hydroxytryptamine
LEP: leptin
INS: insulin
T4: thyroxin
SS: somatostatin
GLP-1: glucagon-like peptide 1
AgRP: Agouti gene-related protein
POMC: proopiomelanocortin
IGF-1R: insulin like growth factor-1 receptor
GHRL: Ghrelin
5-HT4R: 5-hydroxytryptamine receptor 4
GHR: growth hormone receptor
CCK: cholecystokinin
SERT: serotonin transporter
INSR: insulin receptor
GLP-1R: glucagon-like peptide 1 receptor
GSH-PX: glutathione peroxide
GST: glutathione S-transferases
T-AOC: total antioxidant capacity
SOD: superoxide dismutase
MDA: malondialdehyde
NO: nitric oxide
TNOS: total nitric oxide synthase
IUGR: intrauterine growth retardation
C4: complement 4
ROS: reactive oxygen species
TLR4: toll-like receptor 4
MyD88: myeloid differentiation primary response 88
Nrf2: nuclear factor like-2
HO-1: heme oxygenase 1
NQO1: NAD(P)H dehydrogenase 1
Akt: serine-threonine protein kinase
mTOR: mechanistic target of rapamycin kinase
4EBP1: eukaryotic translation initiation factor 4E binding protein 1
P70S6K: ribosomal protein S6 kinase beta-1.
